# Antimicrobial Peptides: Current Status, Mechanisms of Action, and Strategies to Overcome Therapeutic Limitations

**DOI:** 10.3390/microorganisms13112574

**Published:** 2025-11-12

**Authors:** Seong Hwan Kim, Yu-Hong Min, Min Chul Park

**Affiliations:** 1 Department of Pharmacy, Inje University, Gimhae 50834, Republic of Korea; ksh041599@gmail.com; 2 College of K-Bio Health, Daegu Haany University, Gyeongsan 38610, Republic of Korea; yhmin@dhu.ac.kr; 3 Inje Institute of Pharmaceutical Sciences and Research, Inje University, Gimhae 50834, Republic of Korea

**Keywords:** antimicrobial peptide, scaffold, bioengineering technology, lipidation, chimeric recombinant

## Abstract

Antimicrobial peptides (AMPs), evolutionarily conserved components of the immune system, have attracted considerable attention as promising therapeutic candidates. Derived from diverse organisms, AMPs represent a heterogeneous class of molecules, typically cationic, which facilitates their initial electrostatic interaction with anionic microbial membranes. Unlike conventional single-target antibiotics, AMPs utilize rapid, multi-target mechanisms, primarily physical membrane disruption, which results in a significantly lower incidence of resistance emergence. Their broad-spectrum antimicrobial activity, capacity to modulate host immunity, and unique mechanisms of action make them inherently less susceptible to resistance compared with traditional antibiotics. Despite these advantages, the clinical translation of natural AMPs remains limited by several challenges, including poor in vivo stability, and potential cytotoxicity. Bioengineering technology offers innovative solutions to these limitations of AMPs. Two techniques have demonstrated promise: (i) a chimeric recombinant of AMPs with stable scaffold, such as human serum albumin and antibody Fc domain and (ii) chemical modification approaches, such as lipidation. This review provides a comprehensive overview of AMPs, highlighting their origins, structures, and mechanisms of antimicrobial activity, followed by recent advances in bioengineering platforms designed to overcome their therapeutic limitations. By integrating natural AMPs with bioengineering and nanotechnologies, AMPs may be developed into next-generation antibiotics.

## 1. Introduction

Since antibiotics were first used in medical treatment in the 1940s, they have been widely used not only for treating infection diseases, but also in animal husbandry, food production and other various fields. However, the excessive use of antibiotics has led to a severe problem: antimicrobial resistance (AMR). AMR is one of the most serious global healthcare threats, leading to increasing healthcare costs and higher fatality rates. The World Health Organization (WHO) stated that 700,000 people died because of AMR bacteria in 2019. The number of annual deaths attributable to AMR is projected to rise to 10 million by 2050 [1]. Economic burden is also expected to escalate significantly, resulting in annual healthcare expense of USD 1 trillion by 2050 [2]. Moreover, recent reports noted that AMR pathogens were increased during the Coronavirus disease 2019 (COVID-19) pandemic; for example, the incidence of carbapenem-resistant *Enterobacterales* increased from 6.7% in 2019 to 50% in April 2020 [3,4]. Addressing this threat urgently requires the development of novel antibiotics or alternative therapeutic agents. However, antibiotic discovery has steadily declined since 1970s, and only a few antibiotics were developed and introduced to the clinical market for two decades, such as cefiderocol and omadacycline [5]. Hence, there is a critical and unmet need for new antimicrobial agents against drug-resistant bacteria.

Mechanisms of AMR have been reported. For example, β-lactam antibiotics act by inhibiting bacterial cell wall synthesis through binding to and inactivating penicillin-binding proteins (PBPs), which catalyze the cross-linking of peptidoglycan [6]. AMR to β-lactams can arise via production of β-lactamases, which hydrolyze the β-lactam ring and render the drug ineffective [7]. Furthermore, bacteria can acquire resistance by producing altered PBPs that bind the antibiotics with significantly reduced affinity for β-lactams [8]. These mechanisms highlight the limitation of conventional antibiotics that depend on a single-target mechanism of action, as a single modification in the target protein can be sufficient to confer resistance [9]. This challenge is further exacerbated by the emergence of “superbugs” like hypervirulent *Klebsiella pneumoniae* (hvKP), a WHO critical priority pathogen [10]. In these strains, high virulence is converging with multidrug resistance and extensive drug resistance, primarily driven by horizontal gene transfer. The convergence of virulence and resistance in ESKAPE pathogens underscores the urgent need for therapeutic alternatives that can bypass these conventional resistance mechanisms [10]. By contrast, antimicrobial peptides (AMPs) exhibit efficacy against AMR bacteria by acting on multiple targets of the bacterial cell membrane [11]. Therefore, AMPs hold potential to replace or complement conventional antibiotics in the fight against AMR.

AMPs are micro-molecules that serve as natural host defense mechanisms in living organisms. These molecular defenders destroy bacteria through two primary mechanisms: membrane disruption and intracellular targeting [9,12,13]. They initially interact electrostatically with bacterial membranes, creating destabilizing pores that cause rapid cell death. Unlike conventional antibiotics that target specific metabolic pathways, AMPs can penetrate cells and simultaneously interact with multiple intracellular targets, inducing cell damage such as the inhibition of cell wall, protein and nucleic acid synthesis and the activity of enzymes. This mixed multi-hit mechanism makes AMPs more versatile and hinders the development of bacterial resistance, making them inherently less susceptible to resistance compared to conventional antibiotics [12,13]. AMPs also demonstrate remarkably rapid bactericidal effects, destroying target pathogens within minutes and preventing infection progression [14]. These peptides frequently showed synergistic activities in combination with conventional antibiotics [13]. Furthermore, AMPs exhibit a broad-spectrum of activity against fungi, viruses and protozoa as well as bacteria [15,16,17]. Thus, AMPs represent a promising alternative to conventional antibiotics to address the problem of antibiotic resistance.

In addition to antimicrobial activities, they possess various biological functions such as immune-modulatory, wound healing, anti-inflammatory, angiogenesis, and anti-cancer activities [9]. AMPs have already been used in different fields such as agriculture, aquaculture, and the food industry, as well as in therapeutic applications [18]. Recently, AMPs have been applied in the clinical field, such as in the modulation of immune response, treatment of wound healing, and inflammation [14]. Nevertheless, native AMPs have several limitations, including in vivo stability and potential cytotoxicity, for therapeutic application [19]. To overcome AMR of conventional antibiotics and the therapeutic limitations of AMPs, bioengineered AMPs, encompassing chimeric recombinants and chemical modifications, have emerged as a strategy to enhance AMPs’ therapeutic properties, including in vivo stability, toxicity, and pharmacokinetics [20,21,22].

## 2. Antimicrobial Peptide

**Figure 1:**
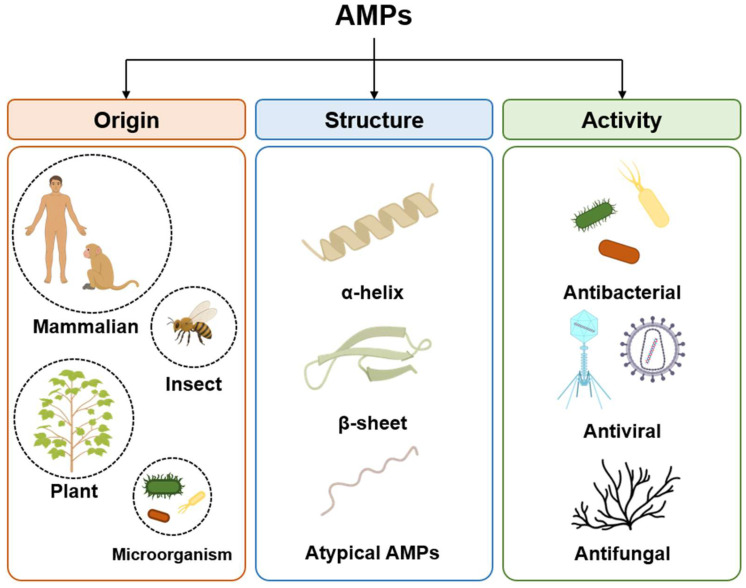
Classification of antimicrobial peptides (AMPs) based on origin, structure and activity. Origin, categorized into mammalian, insect, plant and microorganism sources; structural characteristics, distinguished as α-helices, β-sheet and unique folds; antimicrobial activities, categorized by their primary targets, including bacteria, viruses, and fungi.

AMPs, which are host defense peptides, are short proteins comprising fewer than 100 amino acid residues and that exist in most organisms, such as insects, plants, and animals [23]. They serve important immune roles with defense against bacteria, fungi, and viruses that threaten the host. As AMPs are expressed ubiquitously in various organisms, they comprise numerous types and can be classified according to various criteria, including their origins, structures, and activity (Figure 1) [24,25].

### 2.1. Classification of AMPs by Origin

One of the primary criteria for classifying the diverse types of AMPs is their origin, as they are naturally produced by organisms, contributing distinct structural motifs and biological functions (Table 1). For instance, mammalian AMPs not only exert direct microbicidal effects but also modulate the host immune system; a key example is LL-37, which promotes chemotaxis of immune cells to the site of infection and enhances wound healing [26,27]. In contrast, sessile plants have evolved stable AMPs to defend against pathogens; cyclotides possess a cyclic backbone structure that provides resistance to thermal and enzymatic degradation [28]. According to the antimicrobial peptide database 3 (APD3), 3306 natural AMPs have been identified from various organisms, including human, mammals, amphibians, fish, reptiles, birds, arthropods, insects, and microorganisms [29]. From these diverse sources, this review will focus on representative examples derived from mammals (including humans), insects, microorganisms, and plants.

#### 2.1.1. Mammalian AMPs

Most mammalians, including humans, cattle, and rabbits, have AMPs and use them as defense agents and immune regulators against pathogens. Compared to insect- and plant-derived AMPs, mammalian AMPs possess additional immunoregulatory functions. Owing to their dual roles in antimicrobial defense and immune modulation, mammalian AMPs provide a potent host defense system. This double defense system has driven the evolution of mammalian AMPs beyond simple antimicrobial agents, leading them into multifunctional molecules that bridge the innate and adaptive immune systems while also promoting wound healing [30]. Cathelicidins and defensins are primary families of mammalian AMPs, also known as human host defense peptides. Cathelicidins and defensins are primarily classified based on the presence or absence of the cathelin domain, which is a conserved N-terminal propeptide, approximately 100 amino acids, of cathelicidin [31,32]. While cathelicidin has numerous and various sequences, all mammalian cathelicidins are cationic, amphipathic peptides, a structural feature that disrupts microbial membranes [33]. LL-37 is one of the most well-studied cathelicidins; it is uniquely expressed and secreted by human immune and epithelial cells [27]. LL-37 is the antimicrobial fragment of human cathelicidin protein (hCAP18). LL-37, the C-terminal domain of hCAP18, is composed of a curved amphipathic α-helix structure. LL-37 has +6 positive net charge [34,35]. LL-37 shows broad antimicrobial and antibiofilm activities against Gram-positive and Gram-negative bacteria and exhibits immunoregulatory functions for keratinocytes by activating the signal pathway related to cell survival and growth [36]. The antimicrobial mechanisms of LL-37 are divided into two categories: disruption by interaction with the bacterial membrane and a non-membranolytic mechanism [37]. Specifically, there is an electrostatic interaction between the cationic LL-37 and anionic bacterial membrane, leading to pore formation [37]. Based on an amphipathic α-helix structure, LL-37 penetrates the bacterial membrane and affects the gene expression of the pathogen [38]. The immunomodulatory functions of LL-37 include chemotactic activity on circulating immune cells, the ability to induce pro-inflammatory cytokines from epithelial and circulating cells, and the capacity to modulate pro-inflammatory responses to microbial components [37,39]. Defensins, small and cationic peptides, have a rigid, triple-stranded antiparallel β-sheet structure stabilized by disulfide bonds [40]. α-defensin from human defensins has broad-spectrum antimicrobial potential, including bacteria, fungi, and viruses [41]. Human defensins exhibit an antimicrobial effect by permeabilizing the bacterial membrane and recruiting other components of the immune system to the site of infection [42,43]. Secreted defensins also exhibit chemotactic activity on T cells and immature dendritic cells by interacting with the chemokine receptor CCR6, a crucial mechanism that bridges the innate and adaptive immune responses [44]. Therefore, mammalian AMPs should be considered not merely as direct antimicrobial agents, but also as multifunctional molecules that play diverse and integral roles in host immunity.

#### 2.1.2. Insects AMPs

AMPs derived from insects’ blood cells and fat bodies play a role in survival and defense against bacterial infection [45]. Unlike mammals, insects lack an antibody-mediated adaptive immune system [46]. Instead, they rely on a distinct defense mechanism known as “immune priming”, a process closely linked to the function of their AMPs. In contrast to mammalian AMPs, which act as complex immune modulators, the primary role of insect AMPs is to be rapidly and massively secreted as a diverse cocktail to directly destroy pathogens. This rapid upregulation of AMPs is a key effector mechanism of immune priming [46]. Immune priming protects the insect from lethal infection through the abundant production of AMPs in the hemolymph [46,47]. Pre-exposure of *Galleria mellonella* (*G. mellonella*) larvae to *Candida albicans* protects *G. mellonella* from a subsequent, normally fatal infection. This immune priming was correlated with an increased expression of genes for AMPs with strong antifungal activity, such as gallerimycin and galiomicin [48]. Furthermore, the fat body of insects, a functional equivalent of mammalian liver, produces various AMPs at once, which directly kill pathogens [49]. For instance, when the fruit fly *Drosophila* is attacked by pathogens, 20 different AMPs are produced by the fat body [47]. These AMPs can be classified into several classes, including diptericin (targeting Gram-negative bacteria), attacin (Gram-negative), drosocin (Gram-negative), cecropin (Gram-negative), defensin (Gram-positive), and drosomycin (fungi) [50,51,52,53,54].

#### 2.1.3. Plants AMPs

Plants use the AMPs as a precise immune system to protect against bacteria, fungi, and viruses due to their immovable characteristic. Plants AMPs mostly consist of cysteine-rich motifs and multiple disulfide bonds, including thionins, plant defensins, snakins, and cyclotides [28,55]. A distinguished characteristic of plant AMPs is their remarkable stability, which arises from their unique structural features. They are generally cysteine-rich, enabling the formation of multiple disulfide bonds that establish a rigid tertiary structure. This dense network of cross-links confers remarkable resistance to thermal, chemical, and proteolytic degradation [55]. This is a difference from linear mammalian AMPs such as LL-37, which lack disulfide bonds and are therefore more susceptible to enzymatic degradation. Furthermore, some plant AMPs, known as cyclotides, possess a head-to-tail cyclized backbone. This circular topology provides additional advantages, including resistance to exopeptidase and enhanced thermodynamic stability against unfolding, which contributes to their high rigidity and potent biological activity [56]. Thionins are found in various parts of plants and are composed of 45–47 amino acids, containing 6 or 8 cysteines and 3 or 4 disulfide bonds. Also, they form a cyclic structure like a cyclotide, which is mentioned in 2.1 [56,57]. The stability of plant defense molecules, such as cyclotides, is considered an evolutionary adaptation to the sessile nature of plants, which necessitates a continuous and robust chemical defense system [58]. They exhibit antimicrobial effects against bacteria and fungi, including *Pseudomonas*, *Xanthomonas*, *Erwinia*, *Corynebacterium*, *Thielaviopsis paradoxa*, and *Drechslera teres* [59].

#### 2.1.4. Microorganism AMPs

Microorganism-derived AMPs are produced by bacteria and fungi, with nisin from *Lactococcus lactis* and gramicidin from *Bacillus brevis* being notable examples [60,61,62]. Microorganisms, such as lactic acid bacteria, exist in a constant state of competition with each other. To achieve a competitive advantage, they produce antimicrobial peptides known as bacteriocins [63]. Bacteriocins are ribosomally synthesized AMPs that inhibit or kill closely related bacterial species [63]. These peptides can be classified into several types based on their post-translational modifications [64]. The post-translational modification of nisin involves the NisB-mediated dehydration of serine and threonine residues, followed by NisC-catalyzed cyclization to form lanthionine rings. Nisin’s mechanism of action is initiated by its N-terminal domain, where the A and B lanthionine rings specifically bind to the pyrophosphate portion of Lipid II. This interaction forms a pyrophosphate cage that sequesters the Lipid II head-group, thereby inhibiting peptidoglycan synthesis. Subsequently, the N-terminal domain-mediated sequestering promotes the recruitment of additional nisin molecules and induces membrane insertion to form a pore [65]. In contrast with post-translationally modified nisin, there are bacteriocins that lack post-translational modifications. For example, pediocin-like bacteriocins, which lack post-translational modification, bind with the mannose phosphotransferase system (Man-PTS), which is located for targeting the bacteria membrane and used as a transporter for sugar [66]. Upon binding, these bacteriocins alter the conformation of Man-PTS, leading to the formation of a permanently open pore [64]. This open pore destroys the balance of the intracellular ion balance, resulting in bacterial death. Some bacteriocins interfere with core intracellular processes after entering the cell. For instance, klebsazolicin obstructs the ribosome exit tunnel to suppress protein synthesis [67]. Similarly, lasso peptides Microcin J25 inhibit transcription by interacting with the secondary channel of RNA polymerase following cellular uptake [68]. The diverse antimicrobial mechanisms, observed in microorganism AMPs, are a direct reflection of the evolutionary pressures exerted by their highly competitive environments.

**Table 1:** Classification and characteristics of AMPs by origin.

Origin	AMP Family/ Peptide	Structure	Mechanism of Action	Activity	Characteristics and Function	Reference
Mammalian	Cathelicidins	Cationic, amphipathic peptides	Disrupt microbial membranes	Broad antimicrobial and antibiofilm activity (Gram-positive and -negative)	Possess a conserved N-terminal propeptide (cathelin domain). Performs dual roles in antimicrobial defense and immune modulation	[33]
	LL-37	Curved amphipathic α-helix structure	membrane disruption (pore formation) and non-membranolytic mechanisms	Broad antimicrobial and antibiofilm activity (Gram-positive and -negative)	Immunomodulatory functions	[26,34,35,36,37,38,39]
	Defensins	Cationic peptides, rigid, triple-stranded antiparallel β-sheet structure by disulfide bonds	Increases permeability of bacterial membranes	Antibacterial Antifungal Antiviral	Recruits immune system components to the site of infection. Links innate and adaptive immunity through chemotactic activity on T cells and immature dendritic cells	[40,41,42,43,44]
Insect	Gallerimycin and Galiomicin	Cysteine-rich defensin-like peptides	Induce membrane depolarization	Antifungal	Increased expression during Immune priming in *G. mellonella*	[48,69,70]
	*Drosophila* AMPs	includes various structures	Act by disrupting microbial membranes	Diptericin, Attacin, Drosocin, Cecropin: Gram-negative bacteria Defensin: Gram-positive bacteria Drosomycin: fungi	A cocktail of ~20 different AMPs is secreted from the fat body of *Drosophila* upon pathogen attack	[47,49,50,51,52,53,54,71]
Plant	Thionins	45–47 amino acid, 6–8 cysteines, 3–4 disulfide bonds cyclic structure	Interacts with membrane lipid, leading to increased cell membrane permeability and lysis	Antibacterial Antifungal	Found in various parts of plants High stability	[28,55,56,57,72]
	Plant defensins	Cysteine-rich motifs, multiple disulfide bonds	Interacts with specific membrane components to trigger intracellular signaling cascades that hinder pathogen growth	Antibacterial Antifungal	Found in wheat, barley, etc.	[28,55,73,74]
	Snakins	Cysteine-rich motifs, multiple disulfide bonds	Act by disrupting microbial membranes	Antibacterial Antifungal	Found in potato tubers, etc.	[28,55,73,74,75,76]
	Cyclotide	Head-to-tail cyclized peptide backbone, cysteine-rich with multiple disulfide bonds rigid and stable structure	Disrupts microbial membranes by forming pores or through a detergent-like effect	Antibacterial Antifungal Insecticidal, nematocidal activity	Highly resistant to heat, chemicals and proteases	[28,73,74]
Microorganism	Nisin	Post-translationally modified structure containing lanthionine rings	Binds to Lipid Ⅱ, a precursor for peptidoglycan synthesis, to inhibit cell wall synthesis, and then forms pores in the bacterial membrane	Inhibit or kill closely related bacterial species	Derived from *Lactococcus lactis*	[60,61,62]
	Pediocin-like bacteriocins	Lack post-translational modification	Binds to Man-PTS of target bacteria, forming a permanently open pore that disrupts ion balance	Inhibit or kill closely related bacterial species	Uses Man-PTS as a receptor	[64,66]
	Klebsazolicin	Linear 23 amino acid peptide containing four azle heterocycle and an N-terminal lactamidine ring	Blocks the ribosome exit tunnel to inhibit protein synthesis	Inhibit or kill closely related bacterial species	Acts intracellularly	[67]
	Micocin J25	Lasso peptide	Enter the cell and inhibits transcription by interacting with the secondary channel of RNA polymerase	Inhibit or kill closely related bacterial species	Acts intracellularly	[68]
	Gramicidin	Linear pentadecapeptide with alternation L- and D-amino acids. Forms a helical dimer that functions as an ion channel	Forms a transmembrane ion channel via head-to-head dimerization	Inhibit or kill closely related bacterial species	A representative AMP derived from *Bacillus brevis*	[63,64,65,77,78,79]

### 2.2. Classification of AMPs by Structure

The biological activities and antimicrobial mechanisms of AMPs depend on their structural characteristics. Natural AMPs generally range from 10 to 100 amino acid residues in length, with the majority being shorter than 50 amino acid residues [80]. While several classification schemes exist, this review will categorize AMPs based on several major structural characteristics, including net positive charge, amphiphilicity, α-helical, β-sheet, and atypical structure.

#### 2.2.1. Net Positive Charge and Amphiphilicity

A representative common structural property of AMPs is their net positive charge, which arises from an abundance of basic amino acids such as lysine and arginine, and to a lesser extent, histidine [80,81]. The net positively charged domains of AMPs interact electrostatically with the negatively charged outer layer of bacterial membranes. These interactions destabilize and disrupt membrane integrity through charge-based binding. The amphiphilic property of AMPs is another common structural feature. When hydrophilic and hydrophobic amino acids are spatially segregated within the peptide, it exhibits both hydrophilic and hydrophobic interactions, enabling effective membrane binding and disruption [82]. Hydrophobic residues are particularly critical for antimicrobial activity, as insufficient hydrophobicity weakens membrane attachment and thereby reduces antimicrobial efficacy [83]. However, excessive hydrophobicity can lead to toxicity in mammalian cells [84].

#### 2.2.2. α-Helix

α-helical AMPs represent the most abundant and well-studied classes of antimicrobial peptides, with examples including cecropin, magainin, pleurocidin, melittin, and moricin [85,86]. Typically, these peptides are unstructured in aqueous solutions but adopt a distinct α-helical conformation upon exposure to membrane or membrane-mimicking environments such as liposomes or phospholipid vesicles [87]. The hydrophobic domain of α-helix is crucial for their membrane-targeting activity and the subsequent disruption of the bacterial membrane [88]. α-helices that are entirely hydrophobic are often found embedded within the cell membrane. The hydrophobic side chains interact with the lipid tails of the membrane, anchoring the AMP in place [88]. The antimicrobial activity of α-helical peptides is enhanced by C-terminal amidation. This modification increases the net positive charge of the peptide by neutralizing the C-terminal carboxyl group, which strengthens the electrostatic interaction with the anionic bacterial membrane. In turn, this stabilizes the active α-helical conformation of the peptide at the membrane interface [81,89]. The α-helical peptides are rich in leucine, alanine, glycine, and lysine [68].

#### 2.2.3. β-Sheet

AMPs with a β-sheet structure are typically characterized by two or more β-strands arranged into a sheet, forming a β-hairpin motif [90]. A hallmark of this class is the presence of two to eight cysteine residues that form one to four intramolecular disulfide bonds, which are critical for stabilizing the peptide’s structure and determining its biological activity [91]. For example, disulfide bonds in defensin present structural stability and reduce proteolytic degradation [92]. Therefore, the substitution of the cysteine residues responsible for disulfide bonds leads to a loss of function, demonstrating the essential role of the folded, disulfide-stabilized conformation [93]. β-sheet AMPs are usually amphiphilic, with many β-chains to form hydrophilic and hydrophobic surfaces [93]. In contrast to many α-helical AMPs, β-sheet AMPs typically maintain a stable, pre-formed structure. Consequently, their conformation does not change significantly under the membrane environment [94]. For β-sheet AMPs, the arrangement of abundant cationic residues and hydrophobic side chains on the antiparallel β-sheet scaffold is crucial for their antimicrobial activity [95]. This spatial segregation of charged and nonpolar residues creates a distinct amphipathic surface. This amphiphilicity is essential for the peptide’s function, as it allows the cationic face to interact with the anionic bacterial membrane while the hydrophobic face drives insertion into the lipid core, leading to membrane disruption. Protegrin-1, thanatin, tachyplesin, polyphemusin Ⅰ and gomesin are prominent examples of β-sheet containing AMPs [96,97,98,99,100].

#### 2.2.4. Atypical Structure

Atypical AMPs, which do not contain α-helix and β-sheet structures, are known as non-αβ AMPs, extended or loop peptides. They are classified as tryptophan-rich and proline-rich [82]. Tryptophan-rich peptides, like indolicidin, adopt an amphipathic conformation. A defining feature of indolicidin is its central domain, which is rich in tryptophan residues [101]. This tryptophan-rich region is responsible for the peptide’s affinity for bacterial membranes and contributes to its overall amphipathic character, which is essential for its antimicrobial function [102]. When bound to dodecylphosphocholine micelles, indolicidin adopts a distinct structure. This conformation is stabilized by a central hydrophobic core, which is formed by the interaction of its tryptophan and proline residues. The positively charged termini are placed on the exterior by this conformation and interact with the micelle’s head groups. Based on this conformation change, indolicidin interacts with and disrupts the bacterial membrane [102]. Likewise, the tryptophan-rich region of lactoferricin B adopts an irregular, non-αβ conformation with an extended backbone upon interacting with sodium dodecyl sulfate micelles [103]. Proline-rich peptides have 15–39 residues and are a unique class of AMPs that act on intracellular targets [104,105,106,107]. Recent studies have demonstrated that proline-rich AMPs adopt an extended polyproline-type conformation after being actively transported into the cytoplasm. This rod-like conformation allows the proline-rich AMPs to fit the ribosomal exit tunnel. These physical obstructs inhibit translation by inducing conformation change in the ribosome and then preventing the binding of aminoacyl-tRNA to the ribosome A site, and the polypeptide chain from elongation [108].

### 2.3. Classification of AMPs by Activity

While the APD3 classifies AMPs into 18 distinct categories based on their biological activity, this review will focus on three primary functions: antibacterial, antiviral, and antifungal activities.

#### 2.3.1. Antibacterial Activity

The antibacterial activity of AMPs is primarily based on their cationic and amphipathic structure. The net positive charge facilitates the initial electrostatic attraction to the negatively charged components of bacterial membranes, such as lipopolysaccharides (LPSs) in Gram-negative bacteria and teichoic acids in Gram-positive bacteria [108]. The cationic nature of most AMPs provides selectivity by attracting them to the negatively charged components of bacterial membranes, a feature largely absent in the membranes of mammalian cells [107]. Following this binding, the amphipathic structure—characterized by the spatial segregation of hydrophobic and hydrophilic residues—enables the peptide to insert into and disrupt the lipid bilayer, leading to pore formation [109]. This selective membrane-disrupting mechanism enables many AMPs to exhibit broad-spectrum activity against both Gram-positive and Gram-negative bacteria, including multidrug-resistant strains such as vancomycin-resistant enterococci and methicillin-resistant *Staphylococcus aureus* (MRSA) [25].

#### 2.3.2. Antifungal Activity

The growing resistance to conventional antifungal agents, targeting enzymes, has prompted the exploration of alternative therapeutic pathways. Some of these emerging strategies act on distinct but essential cellular components [110]. For example, T-2307 targets mitochondrial function, leading to the collapse of fungal energy metabolism. Similar to these alternative antifungal pathways and similar to antibacterial AMPs, many antifungal AMPs also kill fungi by disrupting their cell membrane [110]. This process is initiated by the electrostatic interaction between the cationic peptide and the negatively charged fungal membrane, which is based on the high surface content of phosphorylated mannosyl residues in the cell wall and anionic phospholipids, such as phosphatidylinositol and phosphatidic acid, on the membrane [111,112]. This initial electrostatic attraction promotes the hydrophobic insertion of AMPs into the fugal membrane, subsequent membrane disruption, and fungal cell death [111,112]. The fungal cell wall is composed of chitin, which is essential for maintaining cell integrity [68,111]. Several antifungal AMPs, such as nikkomycins and polyoxins, function through a chitin inhibition mechanism, specifically by acting as competitive inhibitors of chitin synthase [111]. The integrity of the fungal cell wall relies not only on chitin but also on other critical components, such as β-glucan. Enzymes like β-glucan synthase are essential for the synthesis of this polymer. Cyclic lipoproteins, such as Daptomycin, inhibit β-glucan synthase non-competitively, leading to cell wall destabilization, increased susceptibility to osmotic stress, and eventual cell lysis [111]. Importantly, chitin and β-glucan do not exist in mammalian cells, making the fungal cell wall a highly selective target for antifungal AMPs. Through their diverse antifungal mechanisms, AMPs display broad-spectrum activity against various fungal pathogens, ranging from yeasts like *Candida albicans* to filamentous fungi such as *Aspergillus* species [25].

#### 2.3.3. Antiviral Activity

AMPs inhibit viruses through several distinct mechanisms: (1) They directly target the viral envelope. AMPs such as indolicidin bind to the viral envelope, inducing membrane instability. This disrupts the virus’s structural integrity and inhibits its ability to infect the host cell [113]. (2) They block viral entry into the host cell. AMPs interact with viral components, such as glycoproteins, to prevent them from binding to host cell receptors [114]. For instance, defensins can bind to herpes simplex virus (HSV) glycoproteins, thereby preventing the virus from attaching to its host and subsequent entry [114]. (3) They inhibit viral replication intracellularly. AMPs can work as intracellular components of the host cell to defend against viral infection. NP-1, a defensin, is translocated into the nucleus of the host cell and suppresses HSV type 2 replication by inhibiting viral DNA synthesis [115]. Through distinct mechanisms, AMPs are effective against a wide range of viruses, including enveloped and non-enveloped types of influenza [25]. AMPs also stimulate the host cell immune system, enhancing the clearance of virus-infected cells. For instance, defensins recruit and activate immune cells such as dendrite and T cells to the site of infection, a process that enhances the elimination of virus-infected cells [114].

## 3. Mechanism of Antimicrobial Activity of AMPs

While the antimicrobial mechanisms of AMP are generally killing microorganisms directly by targeting the membrane, some AMPs also act as immune modulators to kill microorganisms indirectly [81]. The antimicrobial mechanisms of AMPs are classified into two main categories: membrane-targeting and non-membrane-targeting. Each of these categories is further subdivided into various specific mechanisms.

### 3.1. Membrane Targeting Mechanisms

AMPs typically exert a net positive charge ranging from +1 to +9 and have a cationic domain [95]. The Gram-negative membrane consists of an inner membrane and an anionic LPS-rich outer membrane. On the other hand, Gram-positive bacteria lack an outer membrane but possess a thick peptidoglycan layer decorated with negatively charged teichoic acids. The cationic charge of AMPs is crucial for their initial electrostatic attraction to these anionic components of the bacterial membrane. This interaction not only enhances the biological activity of the AMPs but also increases their selectivity against bacteria [116]. While this initial electrostatic attraction is a common principle of most membrane-targeting mechanisms, the subsequent events leading to membrane disruption and cell death are diverse.

**Figure 2:**
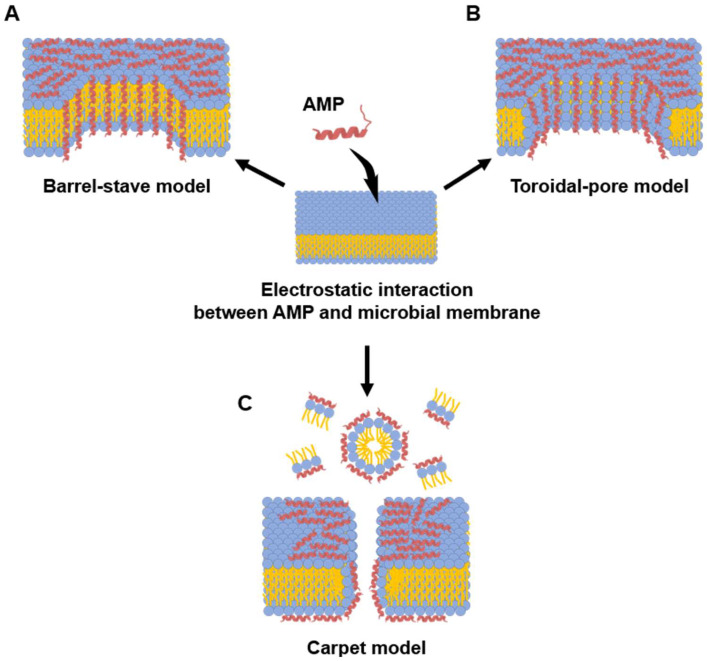
Models of microbial membrane-disrupting mechanisms of AMPs. Membrane interaction of AMPs begins with electrostatic attraction between the cationic peptide residue and the anionic bacterial membrane. (**A**) Barrel-stave model: In this model, AMPs insert perpendicularly into the lipid bilayer. Once embedded, they aggregate and arrange themselves like the staves of a barrel, with their hydrophobic surfaces facing the membrane lipids and their hydrophilic surfaces. (**B**) Toroidal-pore model: This model also involves the perpendicular insertion of AMPs. However, the peptides then induce the lipid monolayers to bend inward, creating a continuous “toroidal” pore that is lined by both the hydrophilic faces of the AMPs and the polar head groups of the phospholipids. (**C**) Carpet model: AMPs accumulate and arrange themselves parallel to the membrane surface. After reaching a critical concentration, they disrupt the membrane in a detergent-like manner, often causing micellization and a complete loss of membrane integrity without forming stable pores. Blue and yellow colored symbol represent hydrophilic head and fatty acid of phospholipid, respectively.

In the barrel-stave model (Figure 2A), amphipathic α-helical AMPs insert perpendicularly into the bacterial membrane [80,85]. Once embedded, these peptides aggregate to form a transmembrane pore [80]. This pore is arranged like the staves of a barrel: the hydrophobic faces of the peptides align with the hydrophobic lipid tails of the membrane, providing stable anchoring, while the hydrophilic faces turn inward to form the lumen of a water-filled channel [92]. This structure results in the unregulated outflow of cytoplasmic contents, including various ions and nutrients [117]. Ultimately, this disruption of homeostasis leads to bacterial death by causing severe ion imbalance and dissipation of membrane potential. Alamethicin and hairpin AMP protegrin-1 perform bactericidal activity through this model [118,119].

While the toroidal-pore model shares similarities with the barrel-stave model (Figure 2B), its defining difference is the involvement of lipids in the pore structure. In this model, after initial electrostatic accumulation, the inserted peptide helices induce a positive curvature in the membrane, causing the lipid monolayers to bend inward continuously. This creates a toroidal or donut-shaped pore where the channel is lined by both the hydrophilic faces of the peptides and the polar head groups of the phospholipids [120,121]. Melittin is a classic example of a peptide that utilizes this mechanism, along with others such as arenicin and magainin 2 [118,121,122].

In the carpet-like model (Figure 2C), after electrostatic attraction of cationic AMPs to the anionic bacterial membrane, the peptides accumulate and arrange themselves parallel to the lipid bilayer, covering the surface like a carpet. Once the peptide concentration on the bacterial membrane exceeds a critical threshold, it disrupts the barrier in a detergent-like manner, leading to membrane permeabilization and the formation of micelles [123]. Because of this mechanism, this model is referred to as the detergent-like model. The human cathelicidin LL-37 is one example of a peptide thought to act through this model [124].

Peptidoglycan is a major structural component of the bacterial cell wall and is a representative feature of both Gram-positive and Gram-negative bacteria. A distinct mechanism for cell wall-inhibiting AMPs involves targeting Lipid II, an essential precursor for peptidoglycan biosynthesis. By binding to and sequestering Lipid II, these AMPs can effectively inhibit the formation of new cell wall material, which weakens the cell and leads to lysis [125]. For instance, nisin exhibits this activity by binding specifically to the pyrophosphate moiety of Lipid II, thereby inhibiting the transglycosylation step of peptidoglycan synthesis [126].

### 3.2. Non-Membrane Targeting Mechanisms

Some AMPs function by translocating across the bacterial membrane without causing significant disruption and then interfering with essential intracellular processes, such as DNA replication and protein synthesis [55]. Once translocated in the bacterial cytoplasm, these non-membrane targeting AMPs act on various intracellular molecules of bacteria. A well-studied example is buforin II, which penetrates the membrane and subsequently binds to DNA and RNA, thereby inhibiting their functions like DNA replication and transcription [127].

Similar to buforin II, which targets intracellular nucleic acids, indolicidin is another AMP that interferes with DNA-related processes. This 13-amino-acid, tryptophan-rich peptide acts by crosslinking single- or double-stranded DNA at abasic sites and by inhibiting the enzyme DNA topoisomerase I [128]. As exemplified by buforin II and indolicidin, intracellular AMPs can disrupt nucleic acid function through multiple mechanisms; they not only bind directly to nucleotides to cause impairment but also inhibit enzymes associated with DNA replication and transcription.

Intracellular AMPs eliminate microorganisms by targeting microbial protein translation machinery [66,129]. The proline-rich peptide Bac7 fragment, Bac7_1-35_, interferes with bacterial ribosomes, leading to the inhibition of translation [106]. PR-39, proline and arginine-rich AMP is actively transported into the cytoplasm of *E. coli* [130]. Once inside the cytoplasm, PR-39 not only inhibits protein synthesis but also causes the degradation of proteins required for DNA synthesis. Similarly, other proline-rich AMPs exert antimicrobial activity by binding to ribosomes and interfering with protein synthesis [131]. Furthermore, some proline-rich, insect-derived peptides inhibit bacterial DNA replication by interfering with the protein folding process. Oncocin, a 19-residue proline-rich AMP, penetrates the bacterial membrane without causing lysis. Once inside, it specifically binds to molecular chaperones, DnaK [132]. Through this interaction, Oncocin disrupts proper protein folding, thereby exerting strong antimicrobial activity against Gram-negative bacteria such as *E. coli*, *P. aeruginosa*, and *A. baumannii* [132].

Conventional antimicrobial resistance arises from specific molecular changes, such as modification of a single protein target. In contrast, many antimicrobial peptides circumvent these mechanisms by employing a fundamentally different strategy: targeting the physicochemical properties of the bacterial membrane itself. It is metabolically and evolutionarily more challenging for a bacterium to fundamentally alter its entire membrane structure than it is to acquire a point mutation in a target enzyme. Therefore, due to their non-specific, membrane-disruptive mechanisms of action, AMPs represent a promising therapeutic strategy for overcoming the challenge of multidrug-resistant pathogens.

## 4. Bioengineered AMPs for Therapeutic Application

### 4.1. Clinical Applications and Limitations of AMPs

Dalbavancin, first identified as BI-397 and discovered in the early 1990s, is produced as a semisynthetic modification of a natural product fermented by *Nonomuraea* spp. It shows promise as a lipoglycopeptide antibiotic with a prolonged duration of action and improved effectiveness against Gram-positive bacteria, including MRSA [133]. Regulatory authorities approved Dalbavancin for treating acute bacterial skin and skin structure infections (ABSSSI), with the Food and Drug Administration (FDA) granting approval in 2014, followed by the European Medicines Agency (EMA) in 2015 [133]. Oritavancin, another semisynthetic lipoglycopeptide antibiotic originating from the vancomycin antibiotic family, was developed to combat severe infections caused by Gram-positive bacteria. Similar to Dalbavancin, it received approval for managing ABSSSI in adult patients from both the FDA in 2014 and the EMA in 2015 [134].

Although AMPs exhibit promising broad-spectrum antimicrobial efficacy and overcome antibiotic resistance through bypassing conventional antimicrobial resistance mechanisms, several innate properties of AMPs pose significant challenges to their clinical application. A primary limitation is their susceptibility to degradation and denaturation by host proteases, which results in a short in vivo half-life. While AMPs exhibit remarkable in vitro antimicrobial efficacy compared to conventional antibiotics, they often fail to persist at the target site with therapeutic concentrations, limiting their sustained antimicrobial effect. For instance, the phase Ⅲ clinical trial of Iseganan, Neuprex, and XMP-629 failed to show potent efficacy compared to conventional antibiotics, because of reduced therapeutic concentration in the human body [135]. Moreover, the multi-target mechanism of action that makes AMPs effective against broad-spectrum pathogens can cause adverse effects such as significant host cytotoxicity and immunogenicity [136]. For example, polymyxins, with their broad membrane-disruptive activity, while effective against bacteria, also cause significant adverse effects in the host, most notably nephrotoxicity. This primary toxicity is caused by the interaction of polymyxins with the epithelial cell membranes of the renal tubules, which can lead to acute tubular necrosis and renal failure [136]. These adverse effects present a major hurdle, complicating the evaluation of their safety profile and dose range in clinical adaptation [136].

### 4.2. Bioengineering Technology to Overcome the Limitations of Therapeutic Peptides

**Figure 3:**
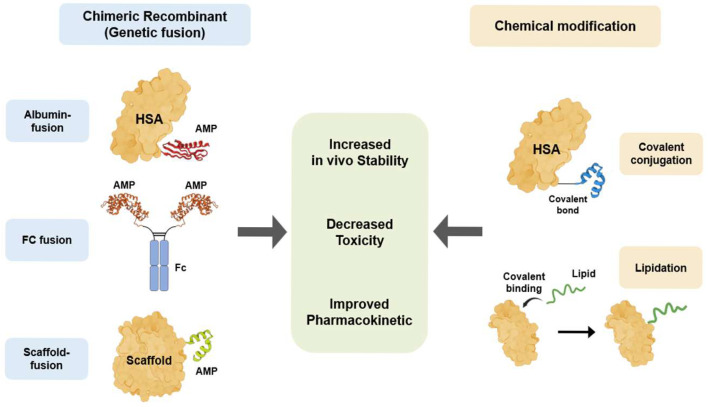
AMP bioengineering methods to overcome therapeutic limitations. Two primary bioengineering strategies have been developed to enhance the therapeutic properties of AMPs. Chimeric recombinant utilizes genetic fusion to link AMPs with stable scaffolds, including HSA, the Fc region of IgG, and other proteins or peptides. In contrast, chemical modification involves the post-synthetic alteration of AMPs, such as the covalent conjugation of the AMPs to HSA or lipidation, in which fatty acid chains are covalently attached to AMPs. Both strategies aim to achieve common therapeutic improvements for AMPs, specifically increased in vivo stability, decreased toxicity, and improved pharmacokinetics.

To overcome the therapeutic limitations of innate AMPs, various bioengineering technologies, including chimeric recombination and chemical modification, have been applied to improve AMPs for therapeutic application [137] (Figure 3 and Table 2). Chimeric recombination is a bioengineering technology that is widely used in protein and antibody therapeutics. This technique involves making a protein by fusing two distinct whole proteins or functional domains to enhance the specific characteristics. For example, a peptide is fused with a stable protein scaffold, such as the Fc domain of an antibody, to improve in vivo stability and half-life [138]. The second bioengineering technique is chemical modification. This strategy involves chemically linking the peptide to another molecule, such as a polymer or a lipid [137]. Chemical modification has been successfully adopted to enhance the stability and half-life of many clinical therapeutics. Chimeric recombination and chemical modification are two powerful bioengineering technologies that are directly applicable to therapeutic AMP development. Using these strategies, researchers can effectively overcome critical limitations such as poor in vivo stability and host cytotoxicity.

#### 4.2.1. Bioengineered AMPs with Human Serum Albumin

In the pursuit of improved biological performance for peptide-based drugs, stable and low-toxicity materials such as albumin have been investigated as a scaffold [139]. Human serum albumin (HSA), in particular, is a natural and highly abundant plasma protein [140]. As the most abundant protein in plasma, HSA is integral to regulating osmotic pressure and pH balance [141]. It also functions as a transporter for numerous insoluble and hydrophobic molecules, including fatty acids, amino acids and hormones [141,142]. A key advantage of HSA is its remarkably long half-life of 19–23 days, which stands in contrast to the much shorter lifespans of most other circulating proteins [141,143]. The stability of HSA is attributed to three primary factors: its internal structure, which exhibits a stable α-helical structure, cross-linked by 17 disulfide bonds; ligand binding, especially fatty acid; and its engagement in neonatal Fc Receptor (FcRn)-mediated recycling [144,145,146]. Therefore, HSA is a promising candidate as a scaffold of chimeric recombination for AMPs, offering a strategy to enhance their limited in vivo stability and reduce immunogenicity. has fusion or conjugation enhances the stability of the partner molecule through steric hindrance, preventing access by proteases, and reducing renal clearance. This technique also reduces the cytotoxicity of the partner molecule by reducing the free form, interacting non-selectively with the host cell membrane.

Chimeric recombinant with HSA enhances peptide or protein by genetically fusing the HSA gene with that of a target peptide or protein, utilizing HSA as a stable and safe scaffold [143]. Through numerous applications of this technology, more than 40 successful clinical trials involving albumin-fused protein drugs have been reported [146]. A key example is albinterferon-α2b, a long-acting interferon developed to treat chronic hepatitis C [147,148]. This fusion protein demonstrates a prolonged half-life of approximately 147 h and extended duration of antiviral activity, compared with the approximately 80 h half-life of non-fused interferon-α2b [149]. Genetic fusion is not the only method to leverage albumin’s long half-life. Chemical modification offers an alternative strategy to attach therapeutic peptides, including AMPs, to albumin [150]. This can be achieved through two main approaches. The first is direct covalent conjugation, which involves chemically linking a peptide to reactive amino acid residues on the surface of HSA. For instance, a drug can be modified with a maleimide group that specifically reacts with the free thiol group of the Cysteine-34 residue on albumin, forming a stable covalent bond [151]. The second, and highly successful, clinical strategy is indirect binding via lipidation. Lipidation induces the non-covalent interaction between the peptide and HSA in the bloodstream [152]. By attaching a lipid moiety (such as a C16 or C18 fatty acid) to an AMP, the peptide gains a strong affinity with HSA [153]. This strategy extends the half-life through a dual mechanism of action. First, once the lipidated peptide enters circulation, it reversibly binds to the albumin protein, which protects it from rapid renal clearance and enzymatic degradation [154]. This complex also benefits from the FcRn-mediated recycling pathway, dramatically extending the peptide’s circulation time from minutes to days [145,146,147]. Second, when administered subcutaneously, these lipidated peptides can self-assemble into multimers, forming a depot at the injection site from which the drug is slowly released into the bloodstream, further prolonging its duration of action [155]. This lipidation strategy has been successful in metabolic diseases, with drugs like liraglutide and semaglutide utilizing this principle to achieve half-lives of 13 h and approximately one week, allowing for once-daily or once-weekly administration [156,157,158]. The clinical success of these drugs demonstrates the profound potential of using lipidation as a chemical conjugation strategy to improve the pharmacokinetic properties of therapeutic peptides, a principle directly applicable to overcoming the stability limitations of AMPs.

Chemical modifications that induce HSA binding can be adopted for AMPs, leading to improvement in in vivo stability and prolonged therapeutic half-life. The peptide FB006M was chemically modified with a 3-maleimidopropionic acid to allow for irreversible conjugation with HSA. This albumin-conjugated FB006M demonstrated a dramatically extended half-life of 10 days, which represents a 120-fold increase compared to that of the unconjugated parent peptide FB006M [159]. HSA-conjugated FB006M exhibits potent antiviral activity against laboratory and clinically isolated human immunodeficiency virus in vitro and in vivo [159]. The antimicrobial peptide K6L9 was chemically conjugated with an albumin-binding molecule (PPA), the stearyl moiety conjugated to the N-terminus of K6L9, to form PPA-K6L9. In a cancer-xenograft mouse model, PPA-K6L9, by circulating as an HSA-bound form, exhibited a significantly enhanced antitumor effect, reducing tumor weight by 90.1% compared to a 47.3% reduction by the control peptide [150]. Furthermore, this albumin-binding strategy significantly reduced systemic toxicities, including hemolysis and hepatic injury, compared to the unmodified AMP [150].

#### 4.2.2. Fc-Fusion Recombinant

Antibodies are another abundant plasma protein. Similar to albumin, antibodies, having a disulfide-bond-based structure, are also stable in vivo. The in vivo half-life of antibody, IgG, in humans typically ranges from 11 to 30 days. Since the Fc region of an antibody governs in vivo stability, the Fc domain is used as a scaffold for chimeric recombination. In the context of AMPs, the goal of Fc fusion, a form of chimeric recombination, is primarily to leverage the Fc region’s extended serum half-life, thereby overcoming the issue of rapid proteolytic degradation that limits the clinical utility of native AMPs [160]. Chimeric recombinant with IgG Fc domain is engineered by fusing a functional peptide or protein to the Fc region of IgG. Unlike native antibodies, Fc-fusion chimeric recombinants lack the light chain of an antibody. Nevertheless, they retain the Fc domain, which confers an extended in vivo half-life. Since Fc-fusion chimeric recombinants prevent non-selective interaction of free-form functional peptides or proteins with host cell membrane, they also exhibit reduced cytotoxicity [161].

Reflecting the success of Fc-fusion chimeric recombination, a number of Fc-fusion protein-based therapeutics have recently received regulatory approval. Notable examples approved by the EMA and FDA include romiplostim (Nplate^®^) [162]. Romiplostim (~60 kDa), for instance, is a dimeric Fc-fusion peptide drug composed of an aglycosylated IgG1 Fc domain produced in *E. coli*, which is fused to two copies of a peptide that mimics thrombopoietin [163]. This Fc-fusion recombinant extends the peptide’s stability in the body, increasing its half-life from a few hours to as long as 139 h [162]. The demonstrated efficacy and safety profiles of these Fc-fusion recombinants have stimulated research into this approach, underscoring its potential as a robust technology for developing next-generation therapeutic molecules [162]. Building on the clinical validation of such Fc-fusion recombinant, this fusion technology represents a promising avenue of research for improving the therapeutic properties of AMPs, particularly to enhance their limited stability and in vivo half-life.

#### 4.2.3. Scaffold-Fusion Recombinant

AMPs can also be fused with other distinct peptides to improve properties such as efficacy, stability, and toxicity. A chimeric recombinant between AMP and a functional peptide combines the advantageous attributes of each parent peptide, aiming to generate a novel hybrid peptide with a broader antimicrobial spectrum, extended stability, administration route, or an improved therapeutic index.

The hybrid gene, generated by genetic recombination, human LL-37 and human beta-defensin (hBD)-129, was synthesized to enhance antimicrobial activity and stability compared to its parent peptides. Against *P. aeruginosa*, the LL37/hBD-129 peptide exhibited a minimal inhibitory concentration of 4 µg/mL, which was 4 times more potent than LL-37 alone (16 µg/mL) and at least 16 times more potent than hBD-129 (>64 µg/mL). Furthermore, the chimeric recombinant showed improved stability against the protease trypsin; after a 120 min incubation, LL37/hBD-129 retained 75% of its antibacterial activity, whereas LL-37 retained only 50% [164]. Another example of a chimeric recombinant is the mussel-adhesive recombinant protein MAP fp-151, which is fused with a functional peptide. MAP fp-151 is a chimera composed of foot protein 1, foot protein 5, and foot protein 1 (fp-1, fp-5, fp-1), derived from the marine organism *Mytilus edulis*. This fusion capitalizes on the robust adhesive and stable properties of the mussel foot protein to potentially anchor the functional peptide at a specific site. MAP was highlighted for its use as a cell and tissue adhesive that promotes cell adhesion and proliferation. For instance, a coating of recombinant MAP was found to significantly improve the adhesion, spreading, and proliferation of osteoblast-like cells. In another example, a fusion peptide containing a MAP fragment and an RGD motif not only promoted the adhesion and spreading of osteoblasts but also activated key factors for osteogenic differentiation [165]. MAP was fused with various AMPs at the C-terminal of MAP fp-151. MAP-AMP chimeric recombinants exhibit enhanced antimicrobial activity against Gram-negative bacteria. Furthermore, MAP-AMP showed remarkable thermal stability at 42 °C for 28 days, and also its antimicrobial activity was sustained. The adhesion properties of the MAP fp-151 chimera make it an ideal scaffold for localized AMP delivery. By fusing the mussel foot protein domain to an AMP, the chimeric recombinant AMPs can be applied as a coating to medical devices or as an adhesive for wound dressings. This application is highly effective for combating biofilm formation, which is a leading cause of chronic infection and medical device failure. The MAP domain anchors the AMP at the site of potential infection, maintaining a high, localized concentration of the antimicrobial agent while simultaneously preventing the rapid systemic degradation and high host toxicity associated with systemic AMP administration [166].

#### 4.2.4. Lipidation

Lipidation, the covalent attachment of lipids to proteins, is a modification that regulates diverse physiological functions by increasing protein hydrophobicity, thereby affecting their trafficking, localization, and stability [167]. This modification typically involves attaching one or more fatty acid chains to the N-terminus or to the side-chains of lysine residues [168]. In the case of AMPs, the incorporation of acyl chains can significantly enhance their therapeutic properties by strengthening membrane interaction and improving resistance to proteolytic degradation, resulting in prolonged half-life and increased efficacy [169,170,171]. The extent of these enhancements is directly correlated with the length of the attached acyl chain; however, a trade-off exists. As the acyl chain length increases, the tendency of the peptide to undergo self-assembly also rises, which can impede the interactions with microbial membranes [172]. Consequently, an optimal acyl chain length is crucial for maximizing antimicrobial activity. For instance, lipopeptides with very long chains (C17 and C20) were inactive, likely due to excessive self-aggregation, whereas optimal activity against bacteria and *Candida* species was observed with shorter fatty acids of C8 to C14 [173]. While the conjugation of fatty acids can increase a peptide’s hydrophobicity and thereby enhance its antimicrobial activity, it is crucial to maintain an optimal hydrophilic–hydrophobic balance to avoid an increase in toxicity [172].

The site of lipidation is also critical. For instance, attaching lipid tails (C5 to C13) to the N-terminus of a Temporin L-derived peptide, [Pro^3^, DLeu^9^, DLys^10^]TL, reduced its net positive charge from +4 to +3, leading to a consequent decrease in activity against *S. aureus*, *K. pneumoniae*, and *P. aeruginosa* [174,175]. In contrast, the para-position of Phe^1^ was identified as the optimal attachment site, as it preserved the +4 charge and demonstrated the highest activity [175]. Furthermore, the conjugation of fatty acids to the synthetic peptide CG117-136 induced a higher α-helical content in a membrane-mimetic environment, which favored enhanced insertion into liposomes and a greater ability to disrupt the bacterial membrane [176]. Lipidation serves a dual purpose in enhancing AMPs; it not only augments their antimicrobial potency but also confers greater stability, resulting in an extended half-life [170,171]. This increased stability is often achieved by either shielding sites susceptible to proteolysis or by promoting the formation of protective supramolecular structures. A case in point is the AMP anoplin, where N-terminal acylation with octanoic, decanoic, or dodecanoic acid imparted significant resistance to enzymatic degradation. Consequently, after a four-hour incubation in a trypsin environment, these lipidated anoplin analogues exhibited 3.5- to 4-fold higher antibacterial activity compared to the unmodified parent peptide [177].

**Table 2:** Bioengineering technologies applicable to AMPs.

Bioengineering Technology	Characteristics	Advantages	Disadvantages/ Challenges	Reference
Albumin-fusion recombinant	Extends half-life by utilizing the FcRn receptor recycling pathway and increasing molecular size to evade renal filtration.	Long in vivo half-life (albumin half-life: approx. 19–23 days)	Low immunogenicity and excellent safety property	[106,130,132,178,179]
Fc-fusion recombinant	Extends half-life by evading lysosomal degradation through the FcRn recycling pathway, similar to albumin.	Long half-life and high stability	A proven platform that has produced numerous blockbuster drugs	[144,145,146,178,180,181,182,183,184]
Scaffold-fusion recombinant	Genetic fusion of two or more functional peptide/protein domains to create a new therapeutic mechanism (e.g., bispecific antibodies, immunotoxins).	Implements new therapeutic paradigms with a single molecule (e.g., linking T-cells and cancer cells)	Increases efficacy and specificity through multi-targeting	[148,185,186]
Lipidation	Extends half-life through a dual mechanism: (1) non-covalent binding to circulating albumin; (2) formation of a depot at the subcutaneous injection site via self-assembly.	Minimal increase in molecular size, which is favorable for preserving drug activity	Utilizes the natural transport protein (albumin)	[151,152,153,154,155,156,187,188,189,190]

## 5. Conclusions

The crisis of AMR, compounded by the stagnant development for conventional antibiotics, necessitates the development of novel therapeutic strategies. AMPs, with their diverse origins, broad-spectrum activity, and unique mechanisms of action—particularly their ability to disrupt microbial membranes—represent a promising alternative to combat multidrug-resistant pathogens.

However, the inherent limitations of natural AMPs, such as poor in vivo stability, short half-life, and potential cytotoxicity, have significantly hampered their translation into clinical practice. This review has highlighted that these challenges are not insurmountable. Advanced bioengineering platforms, specifically chimeric recombination (e.g., with HSA, Fc domain and scaffold peptide) and chemical modification strategies (e.g., lipidation), have emerged as alternatives to overcome these hurdles.

By strategically linking AMPs to larger, more stable scaffolds or moieties, it is possible to dramatically enhance their pharmacokinetic profiles, prolong their circulation time, and improve their therapeutic index. The clinical success of peptide-based drugs like semaglutide, which utilizes similar principles to achieve a long half-life, underscores the viability of these approaches. Therefore, the continued exploration and optimization of these chimeric recombination and chemical modification technologies are critical for transforming AMPs from promising candidates into the next generation of effective and safe therapeutics, offering alternatives in the global fight against antimicrobial resistance.

Furthermore, AMPs can be further integrated into nanocarrier-based or smart delivery systems, enabling controlled activation or site-specific release within the human body. These advanced delivery strategies offer additional protection against proteolytic degradation and reduce off-target cytotoxicity, thereby improving the therapeutic index of AMPs. Moreover, the combination of AMP bioengineering and nanotechnology holds particular promise for overcoming biological barriers, such as biofilms or intracellular infections, that limit conventional antibiotic efficacy. Collectively, these synergistic approaches represent the next generation of AMP therapeutics, integrating molecular-level engineering with precision delivery platforms to achieve potent, safe, and targeted antimicrobial effects.

## Data Availability

No new data were created or analyzed in this study. Data sharing is not applicable to this article.
